# Atypical Bronchial Carcinoid Tumor Revealed by Liver Biopsy

**DOI:** 10.7759/cureus.31104

**Published:** 2022-11-04

**Authors:** Amine Hayoune, Badr Kharouaa, Afaf Thouil, Hatim Kouismi

**Affiliations:** 1 Department of Respiratory Diseases, Faculty of Medicine and Pharmacy of Oujda, Mohammed VI University Hospital, Mohamed First University, Oujda, MAR; 2 Department of Respiratory Diseases, Laboratory of Research and Medical Sciences, Faculty of Medicine and Pharmacy of Oujda, CHU Mohammed VI, University Mohamed First, Oujda, MAR

**Keywords:** atypical, typical, case report, lung cancer, carcinoid tumor

## Abstract

Bronchial carcinoid tumors develop from the Kulchitsky cells of the bronchial epithelium, which are stem cells with neuroendocrine properties. These tumors are divided into two types: typical forms and atypical forms, the latter being much rarer, more aggressive, and having a much higher probability of recurrence and distant metastasis. We report herein a rare case of an atypical lung carcinoid tumor metastatic to the liver. The patient is a 79-year-old woman who presented with purely digestive symptoms evolving for two years, with loss of appetite and deterioration of her general condition. The radiological assessment showed a pulmonary lesion with secondary hepatic and osseous nodules. A hepatic biopsy was performed and morphological and immunohistochemical results were compatible with an atypical bronchial carcinoid tumor, metastatic to the liver and bone.

## Introduction

Bronchial carcinoid (BC) tumors are well-differentiated neuroendocrine tumors with attenuated malignancy [1]. The atypical form of these tumors is much rarer than the typical form [2]. These tumors develop from Kulchitsky cells, neuroendocrine cells normally present in the bronchial mucosa. They are classified into two different histological groups, typical carcinoids (TC) and atypical carcinoids (AC). The distinction between those two entities is based on the presence/absence of necrosis and mitotic activity. Typical carcinoid tumors are generally well differentiated, of low grade, show less than two mitoses/10 high power fields (HPF) and necrosis is often absent. However, atypical carcinoid tumors are of intermediate grade, showing 2-10 mitoses/10 HPFs, and contain areas of necrosis [3]. Bronchial carcinoid tumors are symptomatic in 75% of cases and a paraneoplastic syndrome, called carcinoid syndrome, is found in advanced forms [4]. Herein, we report an atypical BC in a 79-year-old woman who presented with digestive symptoms in whom clinical, imaging, and pathological investigations revealed an atypical bronchial carcinoid tumor, metastatic to the liver and bone.

## Case presentation

**Figure 1:**
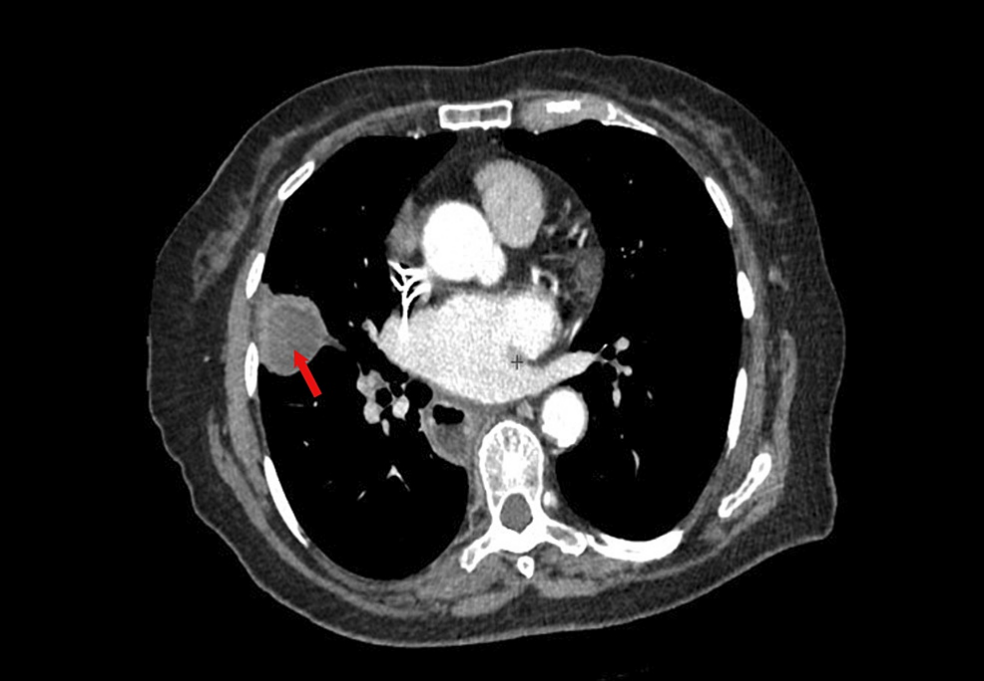
Thoracic CT scan (mediastinal window) showing a 3 x 3 x 2cm spiculated nodule (red arrow) of the right upper lung lobe.

We report a case of a 79-year-old woman, a non-smoker, with a history of treated intestinal tuberculosis. She also had a history of hypothyroidism for two years under treatment (Levothyrox), and cholecystectomy 10 years ago without signs of malignancy on the pathological assessment. She presented to the hospital for intermittent, atypical epigastric pain unrelated to meals associated with vomiting. She reported no jaundice or digestive hemorrhage. The observed epigastric discomfort occurred alongside anorexia and weight loss. There were no reports of flushing, stomach pain, or diarrhea. Clinical examination revealed a dyspneic patient, with epigastric tenderness, and hepatomegaly with a hepatic arrow at 19 cm. No superficial lymphadenopathies were found. Pulmonary auscultation was unremarkable. An abdominal ultrasound was performed and revealed a suspicious gastric wall thickening with multiple secondary liver lesions. Gastroscopy was performed and pathological assessment of the biopsy samples revealed a superficial chronic interstitial gastritis with no malignancy. A thoracic and abdominal computed tomography (CT) scan was performed. It revealed the presence of a suspicious 3 x 3 x 2cm, spiculated nodule of the right lung upper lobe (Figure 1).

**Figure 2:**
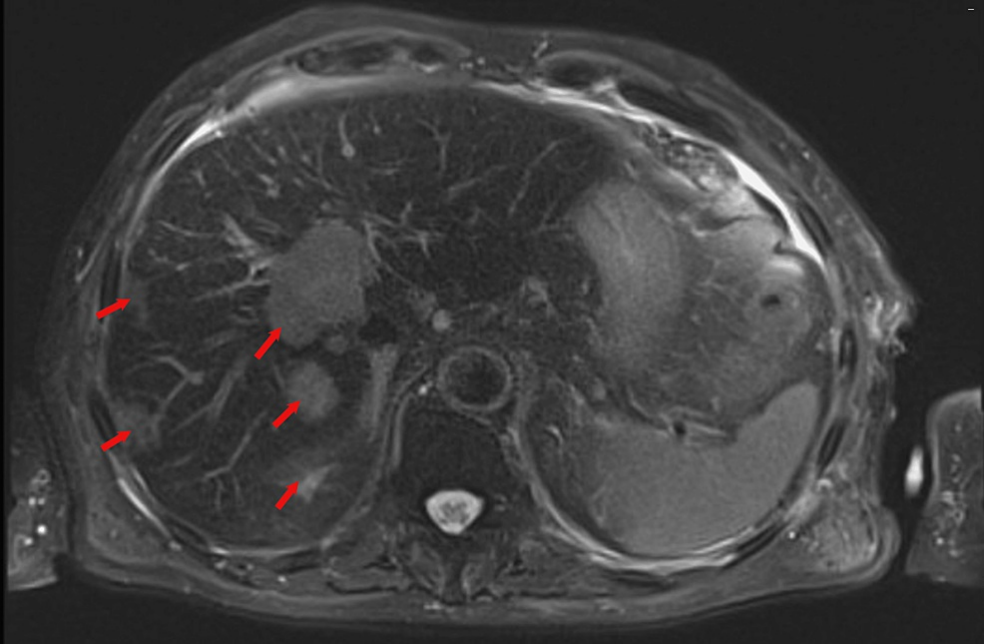
Abdominal magnetic resonance imaging showing the presence of numerous hepatic metastatic nodules (red arrows). The largest one measured 3.5x3x3 cm

On the CT scan of the abdominal level, multiple nodules, the largest of which measured 3.5 centimeters in diameter, were discovered in the liver. These lesions strongly suggested a process of metastasis. Many suspicious lesions were also identified in L1 and L3 vertebral bodies and on the left femoral head. An abdominal magnetic resonance imaging (MRI) was performed and revealed the presence of numerous hepatic nodules measuring between 0.5x0.5 cm and 3.5x3x3 cm. They had irregular contours with no central necrosis. MRI also confirmed the presence of multiple hyperintense nodules in T2 weighted images, on L1, and L3 vertebral bodies, and left femoral head metastatic lesions. These lesions strongly suggested a process of metastasis (Figure 2).

**Figure 3:**
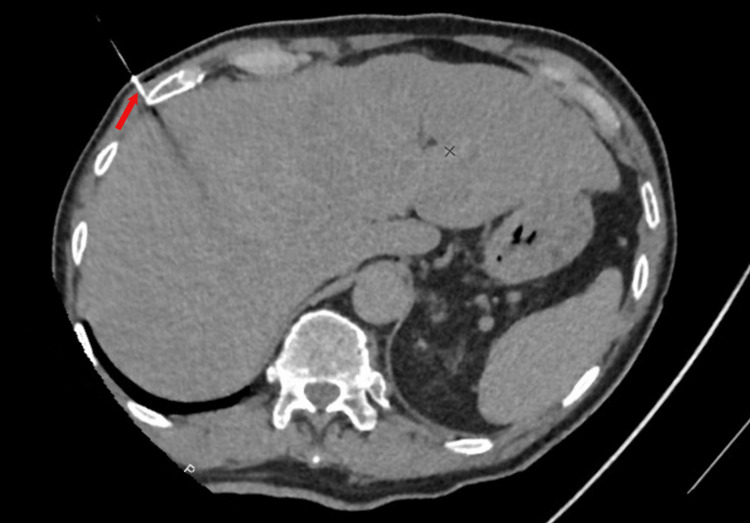
Abdominal CT scan performed to guide the hepatic nodule biopsy. The arrow shows the inserted biopsy needle.

A CT scan-guided liver biopsy was performed (Figure 3).

**Figure 4:**
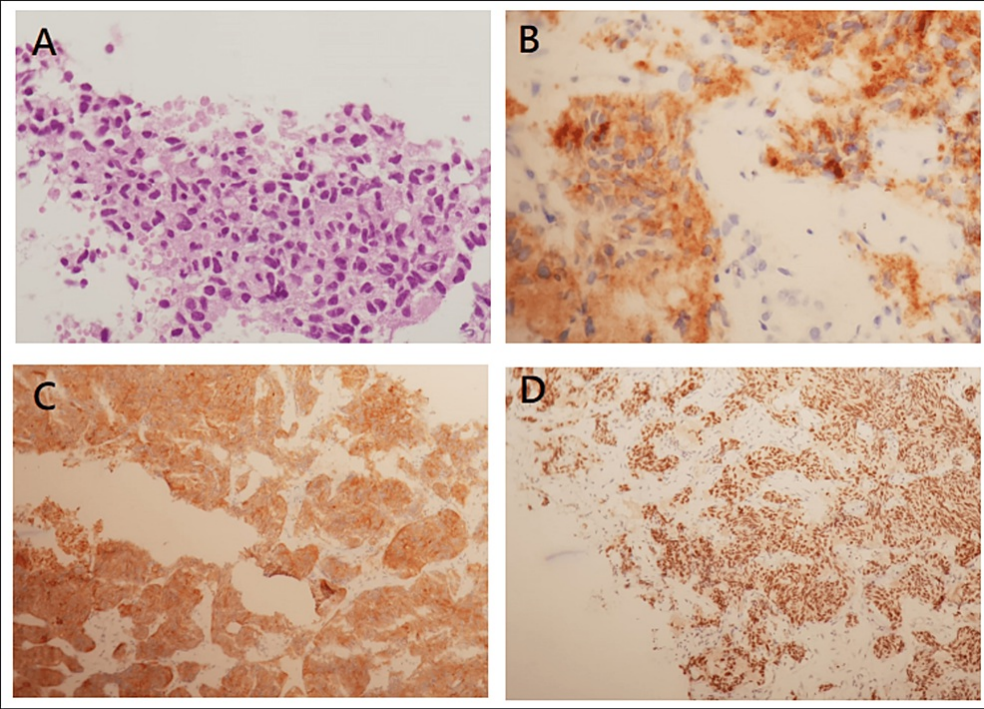
Photomicrograph showing the presence of an epithelial proliferation made of sheets of moderately atypical cells. (HE, 200X) (A). An immunohistochemistry study revealed the expression of chromogranin A (B), synaptophysin (C), and TTF-1 (D) by the neoplastic cells.

The pathological evaluation indicated liver parenchyma infiltrated by an organoid epithelial growth composed of thin sheets and cords of tumor cells. Proliferating cells sometimes showed nuclei with salt and pepper chromatin and some mitotic figures (6 mitoses per 10 high power fields (HFP)). On the neoplastic cells, immunohistochemistry showed the presence of chromogranin A, synaptophysin, and thyroid transcription factor-1 (TTF-1) (Figure 4). Therefore, the diagnosis of atypical bronchial carcinoid of the upper right lung lobe, metastatic to the liver and bones was established.

## Discussion

The neuroendocrine cells found in many organs, including the lung, give rise to neuroendocrine tumors, which are uncommon and heterogeneous. Large cell neuroendocrine carcinoma (LCNEC), atypical carcinoid (AC), typical carcinoid (TC), and small cell lung carcinoma (SCLC) are the four histological variants that make up the heterogeneous family of neoplasms known as neuroendocrine tumors of the lung (Lu-NETs) [5]. Bronchial carcinoid tumors develop from Kulchitsky cells present in the bronchopulmonary mucosa and can secrete hormones, such as adrenocorticotropic hormone (ACTH) or arginine vasopressin, and may therefore cause paraneoplastic syndromes, which resolve with their resection. Their main locations are in the digestive tract (70%), bronchial, ovarian, or even in the thymus. In the American cancer database, carcinoid tumors represent 1-2% of all primary malignant bronchopulmonary tumors in adults. Typical carcinoids (TC) account for 80-90% of all carcinoid tumors. The average age of onset of these tumors is between the third and fifth decades with a peak frequency in the fourth decade [6]. Cases of carcinoid tumors have also been reported in children and adolescents. Smoking cigarettes or being exposed to tobacco smoke is not associated with bronchial carcinoid occurrence [7]. The sex ratio is controversial with studies supporting a balanced distribution between females and males, and others showing a preponderance of atypical carcinoids in men [8,9,10]. Like any neuroendocrine tumor, carcinoid tumors are characterized by the expression of neuroendocrine differentiation markers (chromogranin A, synaptophysin, and CD56) in addition to the expression of TTF-1 [7]. The main reported symptoms for patients with BCs are either bronchopulmonary symptoms linked to the presence of the primary tumor, symptoms linked to metastatic disease - most often bone or hepatic, more rarely secretory syndrome (carcinoid type), fortuitously during systematic assessment as part of the follow-up of a given pathology or in context of multiple endocrine neoplasia type 1 (MEN1). The intensity of reported clinical symptoms depends on tumor location, with symptoms being more numerous and more intense in the case of proximal locations. the clinical presentation is generally variable and may lead to a delay in the diagnosis. Symptomatic patients may present with hemoptysis, chronic cough, chest pain, dyspnoea, and unilateral wheezing which sometimes leads to a wrong diagnosis of asthma or repeated respiratory infections as it can evolve silently with a fortuitous radiological discovery [11, 12]. Rarely, a carcinoid syndrome, especially in metastatic forms, has been reported in some patients. It is characterized by flushing, abdominal cramps, and motor diarrhea. These manifestations are secondary to secretions, typically by a hepatic metastatic carcinoid, of vasoactive substances (serotonin, bradykinin, histamine, prostaglandins, or polypeptide hormones) [11, 13]. A carcinoid syndrome was not reported in our patient despite the secondary hepatic localization. Among lung carcinoid tumors, atypical carcinoids generally present at a more advanced stage than typical carcinoid tumor cases and the risk of loco-regional or metastatic extension is higher: 50-70% of metastatic carcinoid tumors are atypical carcinoids and can appear in many organs and tissues. The main metastatic sites are as follows: liver (75-90%), bone (40-50%), lung (10-40%), and brain (5-15%) [12]. Macroscopically, on the bronchoscopy, this tumor generally presents as a pinkish to reddish mass that may have a polypoid, smooth hypervascularized hemorrhagic appearance but does not represent a contraindication to the performance of biopsies (very low risk of bleeding: less than 1% of cases) [13]. On the radiological level, chest X-rays and CT scans of the thorax can detect these tumors in the form of nodular opacities of central or peripheral location, with the presence of calcifications in 30% of cases or association to lobar or segmental atelectasis. The place of MRI in the diagnosis of these tumors is controversial. It may be indicated when a CT scan study doesn’t assess the vascular or tumoral nature of the lesion [14]. Scintigraphy with somatostatin analogs enables the detection of SST-2 receptors expressed by carcinoid tumor cells. the most widely used marker is octreotide, labeled with indium 111 (Octreoscan®) but is now ideally replaced by 68Ga-DOTA-TOC emission tomography, which provides superior anatomical resolution. The management of isolated pulmonary carcinoid tumors consists of surgical excision, particularly lobectomy with systematic pedicular and ipsilateral mediastinal lymph node dissection taking away the lymph nodes and perinodal fat. The 10-year survival rate reaches 93% in cases of typical and 64% for atypical forms [14]. In patients in metastases stages, the management must be multidisciplinary. It is globally similar to that of metastatic carcinoid tumors of digestive origin and is based on a dual approach requiring on the one hand the control of a possible secretory syndrome when this is present with somatostatin analogs (octreotide or lanreotide) and on the other hand an antitumor treatment [15]. The chemosensitivity of bronchopulmonary carcinoid tumors, although low, is variable. Mono- or polychemotherapy can be performed. Agents such as doxorubicin, 5-fluorouracil (5FU), dacarbazine, cisplatin, etoposide, or streptozotocin showed modest effects [8]. The 5FU-streptozotocin combination is, however, historically the most used combination for atypical carcinoids. Histology is the most important prognostic factor for bronchial carcinoid. Atypical carcinoid tumors have a worse prognosis and a high recurrence rate than CT [16].

## Conclusions

Primary bronchial carcinoid tumors develop from Kulchitsky cells. Primary bronchial atypical carcinoids generally present at an advanced stage compared to typical carcinoid tumor cases and the risk of loco-regional or metastatic extension is higher. Histology is the most important prognostic factor for bronchial carcinoid. As for our case, the diagnosis of atypical carcinoid of the lung should be kept in mind in case of liver metastatic disease.
